# Is There a Tumor Proportion Score Threshold at Which Tumor-Cell PD-L1 Adds Prognostic Information Beyond the International Prognostic Index in Large B-Cell Lymphoma?

**DOI:** 10.3390/diagnostics16183036

**Published:** 2026-09-19

**Authors:** Mehmet Mutlu Kidi, Suheda Atas Ipek, Sendag Yaslikaya, Mahmut Buyuksimsek, Mehmet Turker, Yasemin Aydinalp Camadan, Sedat Biter, Tolga Koseci, Tugba Toyran, Melek Ergin, Berksoy Sahin, Ismail Oguz Kara, Ertugrul Bayram

**Affiliations:** 1 Department of Medical Oncology, Seyhan State Hospital, Adana 01150, Türkiye; 2 Department of Medical Oncology, Faculty of Medicine, Cukurova University, Adana 01330, Türkiye; 3 Department of Medical Oncology, Giresun Training and Research Hospital, Giresun 28100, Türkiye; drysendag@gmail.com; 4 Department of Medical Oncology, Adana Training and Research Hospital, Adana 01330, Türkiye; 5 Department of Medical Oncology, Sanliurfa Training and Research Hospital, Sanliurfa 63300, Türkiye; 6 Department of Medical Oncology, Necip Fazil City Hospital, Kahramanmaras 46080, Türkiye; 7 Department of Medical Pathology, Faculty of Medicine, Cukurova University, Adana 01330, Türkiye

**Keywords:** large B-cell lymphoma, diffuse large B-cell lymphoma, PD-L1, tumor proportion score, immunohistochemistry, prognosis, International Prognostic Index, overall survival, immune checkpoint

## Abstract

**Background/Objectives:** In diffuse large B-cell lymphoma (DLBCL), the prognostic value of programmed death-ligand 1 (PD-L1) depends on the immunohistochemical threshold applied. We asked whether tumor-cell PD-L1 adds prognostic information beyond the International Prognostic Index (IPI). **Methods:** Tumor-cell PD-L1, quantified by the 22C3 tumor proportion score (TPS), was assessed retrospectively in 95 patients with large B-cell lymphoma (87 DLBCL-NOS) given frontline chemoimmunotherapy. TPS was analyzed at the prespecified ≥1% threshold, post hoc at ≥10%, ≥25%, ≥50% and ≥70%, continuously, and with restricted cubic splines. **Results:** PD-L1 positivity (TPS ≥ 1%) was present in 73 patients (77%). After a median follow-up of 89.8 months, PD-L1 was not independently associated with overall survival (OS; adjusted hazard ratio [HR], 0.73; 95% CI, 0.35–1.53) or progression-free survival (adjusted HR, 0.91; 95% CI, 0.44–1.86); no threshold survived correction for multiplicity. An unadjusted association with OS emerged at TPS ≥ 70% (HR, 2.02; 95% CI, 1.02–4.02), but these patients more often had an IPI ≥ 3 (72% versus 36%) and it was attenuated after adjustment (1.53; 0.74–3.18). No definition improved model fit or discrimination beyond the IPI; the null result held in DLBCL-NOS and R-CHOP subgroups. **Conclusions:** Tumor-cell PD-L1 did not add prognostic information beyond the IPI for overall survival.

## 1. Introduction

Diffuse large B-cell lymphoma (DLBCL) is the most common aggressive B-cell non-Hodgkin lymphoma and accounts for approximately 30% of newly diagnosed non-Hodgkin lymphomas [1,2]. It is biologically and clinically heterogeneous: gene-expression profiling separates tumors into germinal-center B-cell-like (GCB) and activated B-cell-like (ABC) subgroups [2], and genomic studies have resolved DLBCL further into genetic clusters whose prognostic impact the 5th edition of the World Health Organization classification considered premature to incorporate [1]. R-CHOP cures more than 60% of patients, but 30–40% develop relapsed or refractory disease with a poor outcome [2]. Biomarkers that refine risk stratification beyond established clinical indices are therefore needed.

The PD-1/PD-L1 axis is a central regulator of peripheral immune tolerance that tumors co-opt to evade host immunity [3,4,5]. Engagement of PD-1 on T cells by PD-L1 restrains T-cell activation, and sustained antigen exposure drives CD8+ T-cell exhaustion; when tumor cells express PD-L1 they suppress effector cytokine release and escape immune surveillance [3,4,5]. Across B-cell lymphomas the extent of this expression varies by entity and shapes susceptibility to checkpoint blockade [5].

In DLBCL, PD-L1 (encoded by *CD274* at 9p24.1) can be upregulated through several routes. Recurrent gains, amplifications and translocations of the *CD274*/*PDCD1LG2* locus correlate with PD-L1 overexpression and are enriched in the non-GCB subtype [6]; structural disruption of the *CD274* 3′-untranslated region stabilizes aberrant transcripts in a further subset [7]; and Epstein–Barr virus-positive DLBCL is likewise enriched for 9p24.1 amplification [8]. Such lesions have been reported in roughly one quarter of DLBCLs, where they mark tumors with a T-cell-inflamed microenvironment [9]. Beyond the malignant compartment, PD-L1 is abundantly expressed by tumor-associated macrophages [10].

The clinical relevance of the axis is supported by the activity of checkpoint inhibitors in PD-L1-driven large B-cell lymphomas. Pembrolizumab produced durable responses in relapsed or refractory primary mediastinal large B-cell lymphoma (PMBCL), where the magnitude of the 9p24.1 aberration correlated with PD-L1 expression and with progression-free survival [11]. In DLBCL, *CD274* alterations have been linked both to inferior progression-free survival after frontline chemoimmunotherapy and to response to PD-1 blockade at relapse [9], so the same feature may carry distinct prognostic and predictive meaning [12].

Despite this rationale, the prognostic value of PD-L1 in DLBCL remains unsettled. Tumor-cell positivity has been reported in approximately 11% of cases and linked to inferior overall survival independently of microenvironmental PD-L1 [13], and to shorter event-free survival elsewhere [4]; other series find an effect only above higher thresholds [3], or report that microenvironmental rather than tumor-cell PD-L1 predicts more favorable outcomes [14]. Interpretation is complicated by methodological heterogeneity: antibody clones, scoring approaches and positivity thresholds differ across studies, and no consensus cut-off has been established [3,15].

Given these uncertainties, and the limited real-world data from our region [16], we retrospectively evaluated tumor-cell PD-L1 expression, quantified as the immunohistochemical tumor proportion score (TPS), in 95 patients with large B-cell lymphoma treated with frontline immunochemotherapy. We asked three questions: whether the prognostic association of tumor-cell PD-L1 depends on the TPS threshold applied; whether the 70% threshold, at which a previous cohort using the same antibody clone reported an association with survival [3], could be reproduced independently; and whether any surviving association adds prognostic information to the International Prognostic Index. The ≥70% threshold was taken from that external report rather than sought in these data. A cut-off both discovered and tested in one cohort cannot be interpreted until examined elsewhere; this study supplies that external test.

## 2. Materials and Methods

### 2.1. Study Design and Patients

This was a single-center, retrospective cohort study conducted at Cukurova University Faculty of Medicine (Departments of Medical Oncology and Pathology), Adana, Türkiye. Consecutive patients with histopathologically confirmed large B-cell lymphoma diagnosed between 2011 and 2022 were identified from the pathology department archive. All cases were reviewed within the framework of the 2022 World Health Organization classification of hematolymphoid tumors, using the available morphological and immunophenotypic findings [1]. The cohort comprised 87 cases of DLBCL, not otherwise specified (DLBCL-NOS), one T-cell/histiocyte-rich large B-cell lymphoma, and seven cases provisionally categorized as high-grade B-cell lymphoma, not otherwise specified (HGBL-NOS). The HGBL-NOS cases were assigned on morphological and immunophenotypic grounds; fluorescence in situ hybridization was not available, so *MYC* and *BCL2* rearrangements were not excluded genetically. In these seven cases the median Ki-67 index was 90% and BCL2 protein was absent in five of the six assessable cases. Because these entities differ in biology and treatment, the prespecified and ≥70% analyses were repeated in the DLBCL-NOS subgroup alone, and separately in patients treated with R-CHOP alone (Section 2.8). Patients whose diagnostic pathology had been performed at an external institution, and patients without regular follow-up records in the institutional electronic medical record system, were excluded; no other exclusion criteria were applied. A total of 95 patients met these criteria and were analyzed. Reporting followed the REMARK recommendations for tumor-marker prognostic studies.

### 2.2. Ethical Approval

The study was approved by the Research Ethics Committee of Cukurova University Faculty of Medicine (Meeting No. 162, Decision No. 58; 9 January 2026) and was conducted in accordance with the Declaration of Helsinki. Owing to the retrospective design, the requirement for written informed consent was waived.

### 2.3. Clinicopathological Variables

Demographic, clinical, laboratory, and pathological data were retrieved from patient records. Recorded variables comprised age at diagnosis, sex, Ann Arbor stage, B symptoms, extranodal involvement, bone-marrow involvement, central nervous system involvement, bulky disease (≥7.5 cm), and the International Prognostic Index (IPI) [17]. Baseline laboratory parameters included lactate dehydrogenase, hemoglobin, albumin, leukocyte count, absolute lymphocyte and neutrophil counts, platelet count, β2-microglobulin, and C-reactive protein. Pathological assessment included the Ki-67 proliferation index (dichotomized at 80%) and CD20 expression.

### 2.4. Immunohistochemical Staining and PD-L1 Scoring

Sections 4 µm thick were cut from formalin-fixed, paraffin-embedded tissue blocks. PD-L1 immunohistochemistry was performed using the 22C3 antibody (pharmDx; Agilent/Dako, Santa Clara, CA, USA) on an automated Ventana BenchMark XT platform (Ventana Medical Systems, Tucson, AZ, USA) with the ultraView Universal DAB Detection Kit (Ventana Medical Systems, Tucson, AZ, USA), using a laboratory-developed, locally optimized protocol. Because the 22C3 pharmDx assay (Agilent/Dako, Santa Clara, CA, USA) is analytically validated on the Dako Autostainer Link 48 (Agilent/Dako, Santa Clara, CA, USA), the present staining should be regarded as a 22C3-based laboratory-developed immunohistochemical evaluation rather than the companion-diagnostic assay. PD-L1 expression was evaluated exclusively as membranous staining of viable tumor cells and was quantified by the tumor proportion score (TPS), defined as the percentage of PD-L1-positive tumor cells among all viable tumor cells. Reactive immune cells served as internal positive controls. Both pathologists reviewed each case together, blinded to clinical data, and recorded a single agreed TPS; no separate scores were retained, and interobserver agreement was therefore not quantifiable.

**Figure 1:**
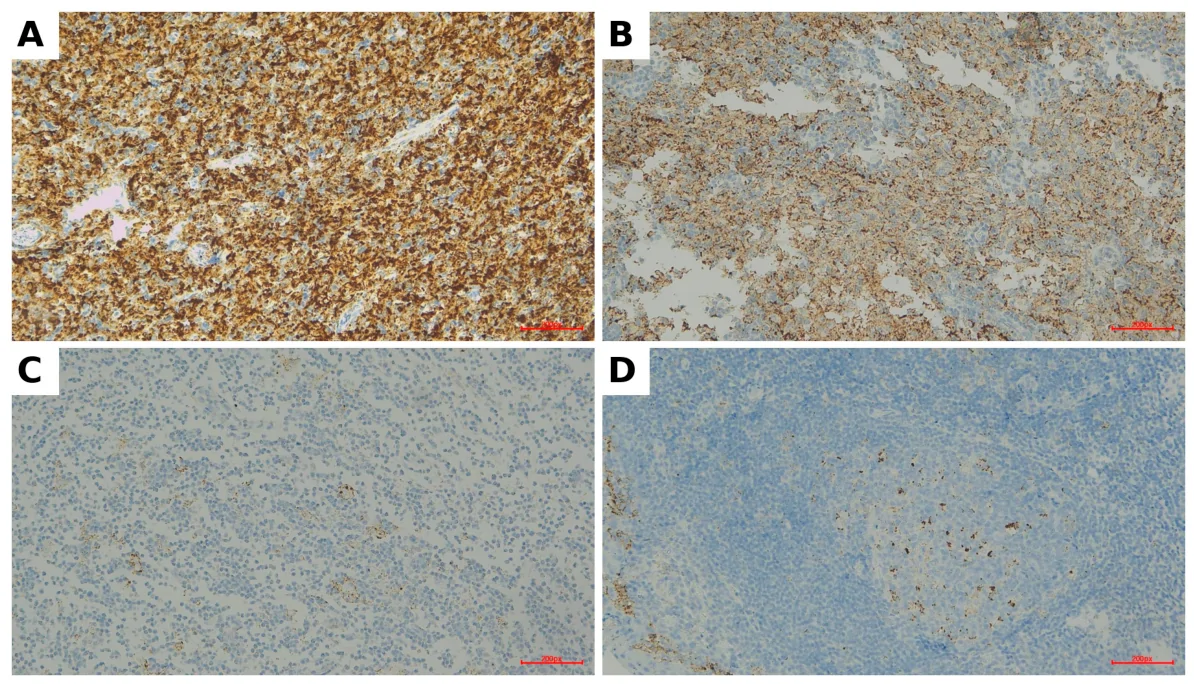
Representative PD-L1 immunohistochemical staining in large B-cell lymphoma (22C3; original magnification 200×). Membranous tumor-cell staining in approximately (**A**) 100%, (**B**) 80%, and (**C**) 5% of neoplastic cells; (**D**) reactive immune cells serving as an internal positive control.

Cases with a TPS ≥ 1% were classified as PD-L1-positive and those with a TPS < 1% as PD-L1-negative; TPS ≥ 1% was the prespecified positivity threshold. Because no consensus cut-off has been established for DLBCL, and because the apparent prognostic value of PD-L1 has been reported to depend on the threshold applied [3,15], additional thresholds were evaluated in post hoc analyses, ≥10%, ≥25%, and ≥50%, together with ≥70%, the threshold at which a previous cohort using the same 22C3 clone reported a significant association with OS [3]. The ≥70% threshold was thus specified from an external report and not selected from the present data. TPS was additionally modeled as a continuous variable (Section 2.8). The distribution of recorded TPS values, and their implications for the operational meaning of these thresholds, is reported in Section 3.2. Representative staining patterns across the TPS range are shown in Figure 1.

### 2.5. Cell-of-Origin and Double-Expressor Assignment

Cell of origin was assigned using the Hans algorithm on the basis of CD10, BCL6, and MUM1/IRF4 immunostaining, classifying cases as GCB or non-GCB [18]. Double-expressor lymphoma was defined as concurrent MYC (≥40%) and BCL2 (≥50%) protein co-expression by immunohistochemistry [19].

Immunostains other than PD-L1 (CD10, BCL6, MUM1/IRF4, CD20, Ki-67, MYC and BCL2) were performed as part of the routine diagnostic workup over the study period, using the antibodies and platforms in clinical use at the time; clone and manufacturer details were not uniformly recorded and could not be retrieved retrospectively for all cases.

### 2.6. Treatment and Response Evaluation

Patients received frontline rituximab-based chemoimmunotherapy, predominantly R-CHOP. Intrathecal methotrexate was administered for central nervous system prophylaxis; systemic high-dose methotrexate was added to the backbone regimen in three patients. Consolidative radiotherapy was given where clinically indicated. Treatment response was assessed by interim and end-of-treatment ^18^F-FDG positron emission tomography/computed tomography (PET/CT) and categorized according to the Lugano criteria [20]. All PET/CT examinations used for response assessment were performed at our institution, on PET/CT scanners from a single manufacturer (Siemens Healthineers, Erlangen, Germany). Primary refractory disease was defined as progression during treatment or within 6 months of its completion. Relapse was documented with its date.

### 2.7. Outcome Definitions

Overall survival (OS), the primary endpoint, was measured from the date of diagnosis to death from any cause. Progression-free survival (PFS), a secondary endpoint, was measured from the date of diagnosis to progression, relapse, or death from any cause, whichever occurred first. Patients without an event were censored at the date of last follow-up (30 April 2026 for surviving patients). Vital status was verified for all surviving patients as of 30 April 2026. Clinical and radiological disease assessment was likewise available through that date for every patient without a progression event, so 30 April 2026 served as the censoring date for both endpoints; no patient was lost to follow-up before that date.

### 2.8. Statistical Analysis

Continuous variables were summarized as median (interquartile range [IQR]) and categorical variables as frequencies and percentages; 95% CIs for proportions were calculated by the Clopper–Pearson exact method. Associations between PD-L1 status and clinicopathological features in the prespecified analysis (Table 1) were examined with the chi-square test with continuity correction, or the Fisher exact test where any expected count was below five, and with the Mann–Whitney U test for continuous variables; in the post hoc comparisons (Supplementary Table S2) the Fisher exact test was applied to all categorical variables. Survival was estimated by the Kaplan–Meier method and compared with the log-rank test; median follow-up was estimated by the reverse Kaplan–Meier method. The prespecified multivariable Cox model included PD-L1 status, IPI, and Ki-67; individual IPI components (e.g., stage, lactate dehydrogenase) were not entered separately to avoid collinearity with the IPI. The results were expressed as hazard ratios (HRs) with 95% CIs; the proportional-hazards assumption was assessed with scaled Schoenfeld residuals against rank-transformed time, multicollinearity with variance inflation factors (VIFs) from intercept-included auxiliary regressions, and model stability with the events-per-variable (EPV) ratio. The prespecified PD-L1 positivity threshold was TPS ≥ 1%. A two-sided *p* < 0.05 was considered statistically significant.

The prespecified analyses were performed in IBM SPSS Statistics version 25 (IBM Corp., Armonk, NY, USA). The post hoc threshold, continuous, spline, and multiplicity analyses were performed in Python version 3.12 (Python Software Foundation, Wilmington, DE, USA) using the lifelines (version 0.30.3) and SciPy (version 1.13.1) packages.

Because the prognostic value of PD-L1 has been reported to vary with the threshold applied [3,15], post hoc analyses were performed: the Kaplan–Meier, log-rank and multivariable Cox analyses were repeated at TPS thresholds of ≥10%, ≥25%, ≥50% and ≥70%, with Benjamini–Hochberg correction within the resulting family of ten analyses; TPS was additionally modeled continuously and with a restricted cubic spline; the incremental value of PD-L1 beyond the IPI was quantified for OS by the likelihood ratio test, Akaike information criterion and Harrell’s *C*-index with bootstrap correction for optimism; and the prespecified and ≥70% analyses were repeated in the DLBCL-NOS and R-CHOP subgroups. No data-driven search for an optimal cut-off was undertaken. Full details, including knot placement, the full range of cut-offs examined, and software versions, are given in the Supplementary Methods.

## 3. Results

### 3.1. Patient Characteristics

A total of 95 patients were analyzed: 87 (92%) had DLBCL, not otherwise specified, one had (1%) T-cell/histiocyte-rich large B-cell lymphoma, and seven (7%) were provisionally categorized as having high-grade B-cell lymphoma, not otherwise specified. Median age was 58 years (IQR, 48–65.5) and 57 patients (60%) were male. Cell of origin was assessable in 50 patients (28 GCB, 22 non-GCB) and double-expressor status in 56, of whom 10 (18%) were positive. CD20 was positive in all cases. All patients received rituximab-based chemoimmunotherapy: R-CHOP in 73 (77%), dose-adjusted R-EPOCH in 20 (21%) and HyperCVAD in 2 (2%); systemic high-dose methotrexate was added in three. The R-CHOP subgroup was defined strictly as the 71 patients who received R-CHOP without added methotrexate. Baseline characteristics are given in Table 1.

### 3.2. PD-L1 Expression and Clinicopathological Associations

The median PD-L1 TPS was 40% (IQR, 5–70; range, 0–100), recorded at 12 discrete levels (0, 5, 10, 20, 30, 40, 50, 60, 70, 80, 90 and 100%). No case was scored between 1% and 4%, so the ≥1% threshold was operationally equivalent to ≥5%; none were between 21% and 29%, so ≥25% was equivalent to ≥30%. At the prespecified threshold of TPS ≥ 1%, 73 of 95 patients (77%; 95% CI, 67–85) were PD-L1-positive; positivity was 72% at ≥10%, 62% at ≥25%, 40% at ≥50% and 26% at ≥70%. Representative staining is shown in Figure 1. PD-L1 status was not associated with any assessed baseline variable, including IPI risk (*p* = 0.48), cell of origin (*p* = 1.00) and Ki-67 (*p* = 0.57) (Table 1); baseline laboratory parameters likewise did not differ by PD-L1 status (Table S4).

### 3.3. Treatment Response

Interim PET/CT was available in 72 patients (complete response in 38, partial response in 21, stable disease in two, progression in 11). At the end of treatment, 66 patients achieved a complete response (69%; 95% CI, 59–79), giving an overall response rate of 78% (74 of 95; 95% CI, 68–86); the remainder comprised partial response in eight, stable disease in one and progression in 19, with one patient not assessable and counted as a non-responder. Primary refractory disease occurred in 19 patients (20%; 95% CI, 12–29), all as end-of-treatment progressions. During follow-up, 44 patients (46%; 95% CI, 36–57) progressed or relapsed and 38 (40%; 95% CI, 30–51) died; every death was preceded by progression or relapse.

### 3.4. Survival and Prognostic Analysis

**Figure 2:**
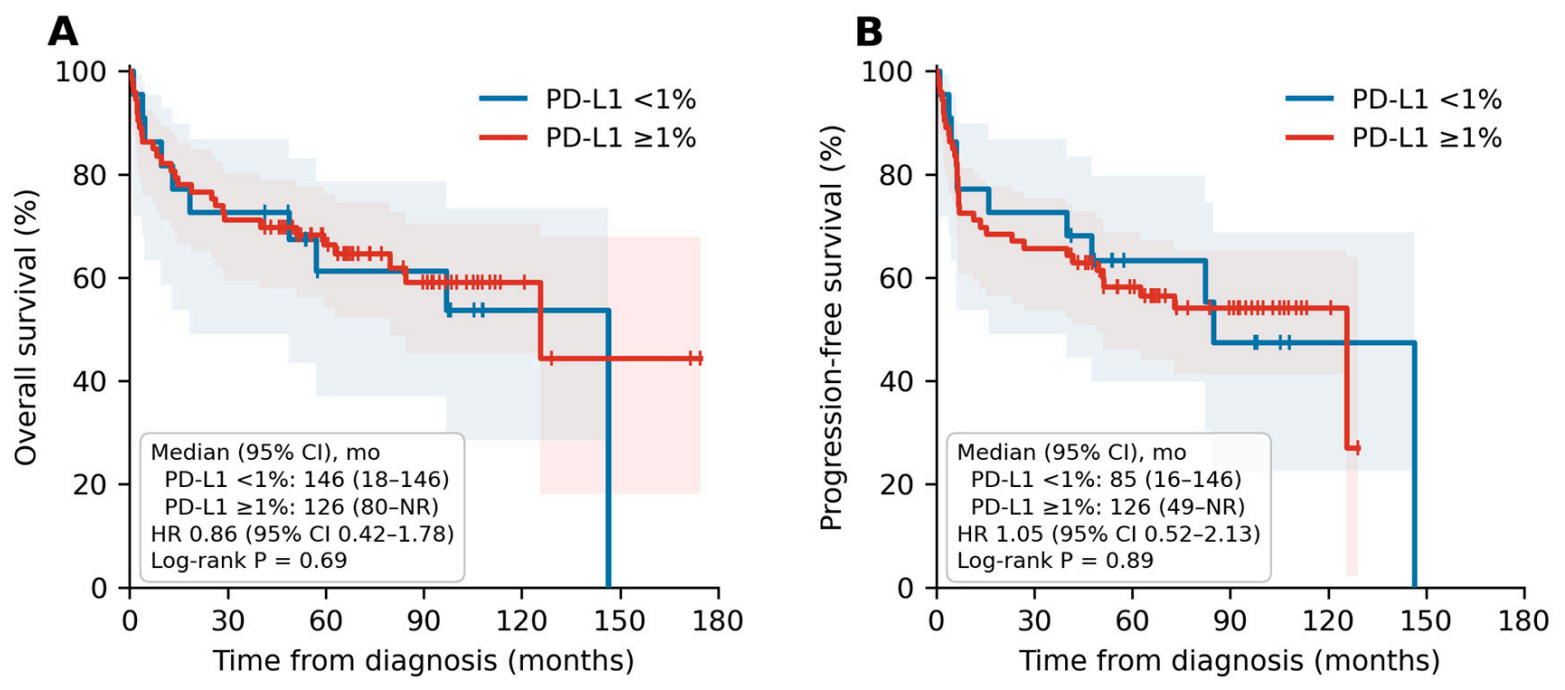
Kaplan–Meier estimates of (**A**) overall survival and (**B**) progression-free survival according to PD-L1 status (TPS ≥ 1% vs. <1%). Shaded bands denote 95% confidence intervals; log-rank *p* values are shown.

After a median follow-up for OS of 89.8 months (95% CI, 66.4–97.6), 38 deaths and 44 PFS events were observed. Median OS was 146.4 months (95% CI, 18.4–146.4) in PD-L1-negative and 125.7 months (95% CI, 79.5–NR) in PD-L1-positive patients (log-rank *p* = 0.69), with 60-month OS of 61% and 67%; median PFS was 84.9 months (95% CI, 16.0–146.3) and 125.7 months (95% CI, 49.2–NR) respectively (log-rank *p* = 0.89), with 60-month PFS of 63% and 58% (Figure 2).

**Figure 3:**
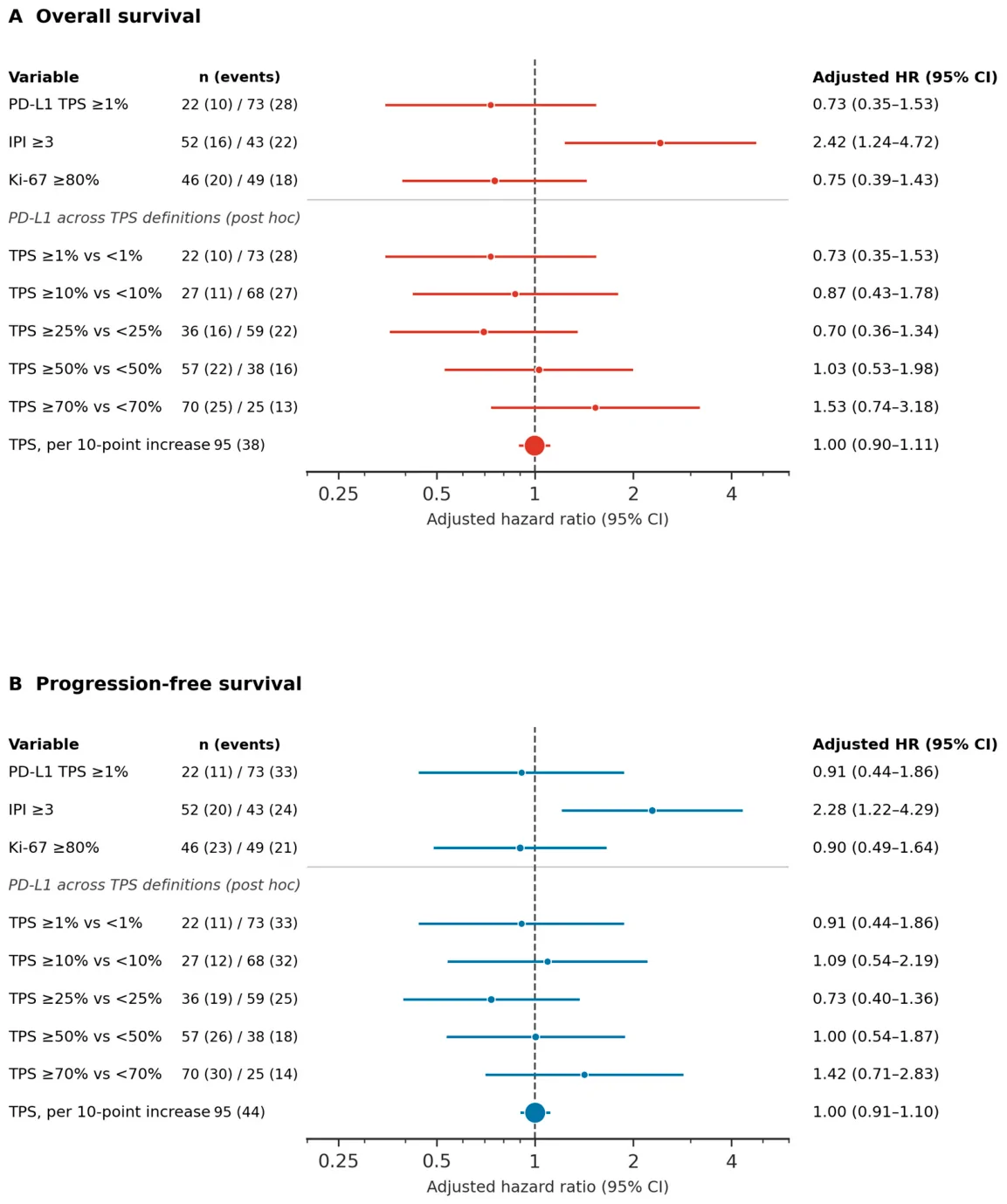
Forest plots of the multivariable Cox proportional-hazards models for (**A**) overall survival and (**B**) progression-free survival. The upper section of each panel shows the prespecified model (PD-L1 TPS ≥ 1%, IPI, Ki-67); the lower section shows the PD-L1 effect across TPS definitions in post hoc analyses, all adjusted for IPI and Ki-67. The ≥70% threshold was specified from an external report [3] rather than from these data. Boxes are proportional to precision, horizontal lines denote 95% CIs and the dashed line marks HR = 1. Rows in the lower section represent the same 95 patients under different definitions of one biomarker and are not independent; no pooled estimate is shown.

On univariable analysis, advanced stage, bone-marrow involvement and IPI ≥ 3 were associated with shorter OS and PFS (Table 2). In the prespecified multivariable model, PD-L1 positivity was non-prognostic for OS (adjusted HR, 0.73; 95% CI, 0.35–1.53; *p* = 0.40) and PFS (adjusted HR, 0.91; 95% CI, 0.44–1.86; *p* = 0.79), while IPI ≥ 3 was the only covariate independently associated with both endpoints (OS: adjusted HR, 2.42; 95% CI, 1.24–4.72; PFS: 2.28; 95% CI, 1.22–4.29) (Table 2; Figure 3). The proportional-hazards assumption was satisfied throughout (Schoenfeld *p* > 0.05), with events per variable of 12.7 (OS) and 14.7 (PFS) and no multicollinearity (variance inflation factor ≤ 1.02).

These analyses were repeated in full at TPS thresholds of ≥10%, ≥25%, ≥50% and ≥70%. PD-L1 was not associated with either endpoint at any of the five thresholds: the adjusted HR ranged from 0.70 to 1.53, every 95% CI included 1.00, nominal *p* values ranged from 0.25 to 0.99 and corrected values from *q* = 0.81 to 0.99. IPI ≥ 3 remained independently associated with both endpoints in all ten models (adjusted HR, 2.05–2.46; all *p* ≤ 0.036). Unadjusted, the log-rank *p* value was 0.497 or higher at every threshold except ≥70% (*p* = 0.041 for OS, 0.061 for PFS), where *q* was 0.30 for both (Table 3; Figure 3). Among the ten distinct partitions produced by every cut-off leaving at least eight patients per group, ≥70% was the only one reaching nominal significance (Supplementary Tables S1A and S1B; Supplementary Figure S2).

At the ≥70% threshold, 25 patients (26%) were PD-L1-positive. Median OS was 146.4 months (95% CI, 96.8–NR) below and 62.9 months (95% CI, 18.7–NR) at or above it, with 60-month OS of 69% and 56% (log-rank *p* = 0.041); median PFS was 125.7 and 41.9 months, with 60-month PFS of 64% and 48% (log-rank *p* = 0.061). Unadjusted, TPS ≥ 70% was associated with OS (HR, 2.02; 95% CI, 1.02–4.02; *p* = 0.045) but not PFS (HR, 1.84; 95% CI, 0.96–3.52). Patients above the threshold more often had an IPI ≥ 3 (72% versus 36%; *p* = 0.002) and advanced stage (88% versus 64%; *p* = 0.039), whereas Ki-67, bone-marrow involvement, B symptoms, bulky disease and cell of origin did not differ (Supplementary Table S2). Adding covariates one at a time, the PD-L1 estimate moved from 2.02 to 1.94 on adding Ki-67, to 1.60 (95% CI, 0.78–3.29) on adding the IPI, and to 1.53 (95% CI, 0.74–3.18) in the full model, in which IPI ≥ 3 remained independently associated with OS (adjusted HR, 2.09; 95% CI, 1.05–4.14; *p* = 0.036).

Modeled continuously, TPS was not associated with OS (adjusted HR per 10-point increment, 1.00; 95% CI, 0.90–1.11) or PFS (1.00; 95% CI, 0.91–1.10). In restricted cubic spline models the overall association was non-significant for OS (*p* = 0.18) and PFS (*p* = 0.37), as was departure from linearity (OS, *p* = 0.06; PFS, *p* = 0.16). Relative to a TPS of 0%, the adjusted HR at 50% was 0.51 (95% CI, 0.22–1.21) for OS and at 100% was 1.38 (95% CI, 0.52–3.67); spline curves are shown in Figure S1.

When Ann Arbor stage was forced into the model, PD-L1 remained non-prognostic for OS (adjusted HR, 0.64; 95% CI, 0.31–1.35) and PFS (0.81; 95% CI, 0.39–1.65). In this model the events per variable fell to 9.5 for OS and the variance inflation factor rose to 1.62; the IPI was no longer independently associated with either endpoint (OS: adjusted HR, 1.31; 95% CI, 0.64–2.67), whereas advanced stage was (OS: 4.71; 95% CI, 1.49–14.85; PFS: 13.93; 95% CI, 3.16–61.42).

### 3.5. Incremental Prognostic Value and Sensitivity Analyses

The contribution of PD-L1 beyond established clinical risk was assessed directly for OS. Entered as its ordinal score, the IPI was associated with OS both alone (HR per point, 1.65; 95% CI, 1.16–2.35) and with Ki-67 (1.64; 95% CI, 1.15–2.35). Under this specification the PD-L1 estimate at TPS ≥ 70% was further attenuated (adjusted HR, 1.43; 95% CI, 0.68–3.02). Adding a PD-L1 term improved fit at no definition: the likelihood ratio test was non-significant in every case (*p* = 0.27–0.99) and the AIC increased throughout (ΔAIC, +0.81 to +2.00). The apparent gain in Harrell’s *C*-index was at most 0.017, and after correction for optimism no *C*-index rose by more than 0.004 above its base model (Table 4). The conclusion was unchanged when Ki-67 was added to the base model (Supplementary Table S5).

Because the cohort included eight patients with large B-cell lymphoma other than DLBCL-NOS and three chemoimmunotherapy backbones, the analyses were repeated in the 87 patients with DLBCL-NOS and in the 71 treated with R-CHOP. The prespecified result was unchanged: the adjusted HR for TPS ≥ 1% was 0.74 (95% CI, 0.34–1.60) in DLBCL-NOS and 0.88 (95% CI, 0.37–2.09) in the R-CHOP subgroup, against 0.73 in the full cohort. At TPS ≥ 70%, restriction strengthened rather than diluted the unadjusted association (DLBCL-NOS: HR, 2.43; 95% CI, 1.18–4.97), but the adjusted estimate remained non-significant in every subgroup (adjusted HR, 1.33–1.67; all *p* ≥ 0.189) (Supplementary Tables S3A and S3B).

## 4. Discussion

In this single-center retrospective cohort of 95 patients with large B-cell lymphoma, tumor-cell PD-L1 assessed by the 22C3 TPS was not independently associated with OS or PFS. Positivity was frequent (77% at TPS ≥ 1%) and unrelated to baseline features. The null result was not confined to that threshold: across five thresholds and both endpoints the adjusted HR ranged from 0.70 to 1.53, every 95% CI included 1.00, and the same held for continuous and spline models. One exception emerged unadjusted: at TPS ≥ 70%, PD-L1 was associated with OS (HR, 2.02; 95% CI, 1.02–4.02), an association examined below. No PD-L1 definition added prognostic information to a model containing the IPI, and the results were unchanged in the DLBCL-NOS and R-CHOP subgroups.

The observed positivity of 77% exceeds that of several previous DLBCL series, in which tumor-cell positivity at the same nominal TPS ≥ 1% threshold was 23.2% [4] and approximately 11% [13]. Threshold choice does not explain the gap: our positivity remained 40% even at TPS ≥ 50%, so our most stringent threshold classified more patients as positive than their most permissive one. The variability reflects differences in antibody clones, staining platforms and the compartment scored [3,15]; when all cell types are assessed, up to 85–95% of de novo DLBCLs are positive, predominantly on myeloid and macrophage populations [21]. Comparability studies show that PD-L1 assays differ in sensitivity and that agreement between them cannot be assumed, with immune-cell scoring the least reproducible component [22]. Our own concern is narrower and does not follow from those data: the 22C3 antibody was applied outside the platform on which it is validated, so our scores are those of a laboratory-developed test rather than the companion-diagnostic assay. “PD-L1-positive” here should therefore not be assumed to denote the same biological population as in series using other assays, a caveat that applies to every cross-study comparison below.

The prognostic role of PD-L1 in DLBCL remains inconsistent. Two meta-analyses reported an association with inferior OS (pooled HR, 2.13; 95% CI, 1.34–3.38 [23]; 1.70; 95% CI, 1.05–2.74 in the DLBCL subgroup [24]), and tumor-cell PD-L1 retained independence in a large clinicopathological series while microenvironmental PD-L1 did not [13]. Others link positivity to shorter event-free survival [4] or to outcome only above higher thresholds [3], whereas microenvironmental PD-L1 has been associated with more favorable outcomes [14], macrophage PD-L1 may shape the immune contexture independently [10], and soluble PD-L1 independently predicted poorer OS [25]. These reports differ not only in conclusion but in what was measured.

A specific consequence of scoring only the malignant compartment deserves emphasis. The TPS counts membranous staining of viable tumor cells and, by construction, discards PD-L1 carried by the immune infiltrate. In DLBCL that infiltrate is not a minor contributor: when all cell types are scored, up to 85–95% of de novo cases are PD-L1-positive, with expression residing predominantly on myeloid and macrophage populations [21], and PD-L1-expressing macrophages suppress anti-lymphoma CD8+ T-cell responses in experimental models [10]. Diagnostic practice in solid tumors has moved for precisely this reason. The combined positive score (CPS), which adds PD-L1-stained lymphocytes and macrophages to the numerator, was developed because the tumor proportion score enriched poorly for response in gastric cancer: at a cut-off of 1%, TPS identified 12.5% of patients with an odds ratio for response of 1.4, against 57.6% and 2.8 for a CPS of one or more, and CPS is now the scoring method used with this antibody in gastric and gastro-esophageal junction adenocarcinoma [26]. Our null result therefore concerns tumor-cell PD-L1 as defined by the TPS, and should not be read as evidence that the PD-L1 axis is uninformative in large B-cell lymphoma. A CPS-type score, or a formal assessment of macrophage-associated PD-L1, might give a different answer; neither was tested here.

The heterogeneity of the disease constrains what any single cohort can establish. WHO-HAEM5 defines the large B-cell lymphoma family as DLBCL-NOS together with more than fifteen further entities, separated by morphology, genetics, viral association, or anatomical site [1]. Several of them—primary mediastinal large B-cell lymphoma, T-cell/histiocyte-rich large B-cell lymphoma, and EBV-positive DLBCL—are precisely the entities in which PD-L1 expression is characteristic rather than incidental [27]. DLBCL-NOS is itself a diagnosis of exclusion, and resolves further into genetic clusters whose prognostic weight the classification considered premature to incorporate [1]. Our cohort was assembled as large B-cell lymphoma and comprised 87 DLBCL-NOS, seven HGBL-NOS, and one T-cell/histiocyte-rich case; although the analyses were reproduced in the DLBCL-NOS subgroup, the conclusion still rests on a biologically heterogeneous group. The same qualification applies to the earlier reports against which we compare our results, few of which were restricted to a single WHO-defined entity. Part of the discordance in this literature may therefore reflect differences in what was aggregated rather than differences in PD-L1 biology.

The single exception requires closer examination. Cin et al. applied the same 22C3 pharmDx antibody to 130 large B-cell lymphomas and reported no difference at a 30% cut-off but a significant association at ≥70% (HR, 2.65; 95% CI, 1.05–6.68) [3]. Their model is ambiguously reported: the covariate table and a single model chi-square suggest a multivariable fit, while the text describes the variables as entered univariately [3]. Read as adjusted, the comparator is our 1.53 (95% CI, 0.74–3.18), an interval containing both their estimate and 1.00, so we neither reproduce nor contradict them; read as unadjusted, it is our 2.02 (95% CI, 1.02–4.02), concordant in direction and magnitude, and our contribution is to show what adjustment does.

What adjustment does is visible in the data. Patients with a TPS ≥ 70% more often had an IPI ≥ 3 (72% versus 36%) and advanced stage (88% versus 64%); adding Ki-67 left the estimate essentially unchanged (HR, 1.94), whereas adding the IPI reduced it to 1.60 (95% CI, 0.78–3.29). Why the same adjustment did not attenuate their estimate cannot be determined from the published report, but their model entered the IPI alongside several of its own components, distributing disease burden across collinear terms; the interval they report for the IPI (1.07 to 20.21) is consistent with this [3]. Our data reproduce that behavior: forcing Ann Arbor stage into our model cost the IPI its independent association (adjusted HR for OS, 2.42 to 1.31) while leaving PD-L1 unchanged, yet the variance inflation factor rose only to 1.62. An IPI term sharing a model with its own components should not be assumed to control for disease burden.

Three further observations bear on the ≥70% signal. It did not survive correction within the threshold family (*q* = 0.30). It was not stable across adjacent cut-offs, our unadjusted HR being 1.06 at ≥60% and 1.60 at ≥80%; threshold behavior was unstable in their cohort too, where the difference was already significant at 60% in the DLBCL-NOS subgroup [3]. And the comparison is not a strict replication at the level of the assay: they used 22C3 pharmDx on the platform on which it is validated and cut 2 µm sections, whereas we used a Ventana BenchMark XT and 4 µm sections. Our assay classified 26% of patients as positive at ≥70% against 12% in their cohort, and 77% versus 48% at ≥1% [3]. The same nominal threshold therefore did not select the same proportion of patients.

The comparison can also be made at a matched threshold. Qiu et al. pooled 12 studies comprising 1478 patients and reported an association with inferior OS (pooled HR, 2.13; 95% CI, 1.34–3.38) that reached significance only among studies using cut-offs of ≥30% [23]. Our ≥25% threshold is operationally ≥30%; at it our unadjusted HR was 0.81 (95% CI, 0.43–1.55) and our adjusted HR 0.70 (95% CI, 0.36–1.34), so a hazard ratio of the pooled magnitude lies outside both intervals. For PFS the picture is agreement: our 1.05 (95% CI, 0.52–2.13) is close to the null pooled estimate of 1.11 [23]. The same meta-analysis suggests why the OS estimates diverge: it found PD-L1 positivity to be more frequent among patients with an IPI of 3–5 (OR, 1.55; 95% CI, 1.11–2.17) [23]—the association we observed—while its pooled estimates were assembled partly from hazard ratios reconstructed from published Kaplan–Meier curves, which are necessarily unadjusted. Such a pooled estimate cannot separate an effect of PD-L1 from the disease burden accompanying it; in our cohort that separation was possible and the association did not survive it (Table 4).

The contrast with PMBCL is instructive, because it is the large B-cell lymphoma in which this axis is best characterized, and it differs from DLBCL-NOS in frequency, intensity, and genetic basis alike. Across 237 aggressive lymphomas assessed by quantitative immunohistochemistry, robust PD-L1 protein was found in the majority of PMBCL and T-cell/histiocyte-rich large B-cell lymphoma, whereas neither the malignant nor the non-malignant cells of EBV-negative DLBCL-NOS showed detectable PD-L1 [27]. The genetic substrate differs to the same degree. In PMBCL the *CD274/PDCD1LG2* locus at 9p24.1 is rearranged in about 20% of cases, specifically so in comparison with DLBCL, follicular lymphoma, and Hodgkin lymphoma, and rearrangement correlates with overexpression of the ligand transcripts [28]. In DLBCL-NOS, 9p24.1 amplification is found in roughly 3.5–4% of cases and copy gains in a further 6–8% [15,29], with genetic lesions of the locus overall in about one quarter [9]. PD-L2 follows the same pattern, detectable in 72% of PMBCL against 3% of DLBCL and, in PMBCL, confined to tumor cells rather than intratumoral macrophages [30]. Two consequences follow. First, in PMBCL, ligand expression tracks a specific and quantifiable genetic lesion, and the magnitude of that lesion correlated with both expression and progression-free survival under PD-1 blockade [11]; in DLBCL-NOS, no such correspondence holds, because immunohistochemical positivity—11% to 77% depending on antibody, platform, and threshold [3,4,13]—exceeds the frequency of any 9p24.1 abnormality several-fold. Most tumor-cell PD-L1 detected by immunohistochemistry in DLBCL-NOS is therefore not driven by the lesion that makes the marker interpretable in PMBCL. Second, PD-1 itself is carried chiefly by the infiltrating T cells rather than by the tumor, so no tumor-cell score captures the receptor side of the axis [5]. A TPS threshold cannot separate these situations, which is a further reason not to expect it to behave as a stable prognostic marker.

The spline models warrant one qualification. Departure from linearity approached significance for OS (*p* = 0.06), reflecting a non-monotonic fitted curve, but we do not read this as a dose–response: the overall test of association was non-significant (*p* = 0.18), every pointwise band included 1.00, and four- and five-knot models did not converge. With 38 deaths and TPS recorded at 12 discrete levels such a model cannot separate a genuine non-monotonic relationship from sampling variation.

Biology offers a second explanation, distinct from confounding: confounding would account for an association that is observed but not causal, and dilution for a real effect that is not observed. The prognostic direction of the 9p24.1 lesion is itself unsettled—amplification has been linked to a PMBCL-like subset with better event-free survival [29] and elsewhere to shorter overall survival [15]. If the lesion that most reliably drives high tumor-cell PD-L1 carries opposite signals across cohorts, a score that cannot identify it is unlikely to carry a consistent one. Our data cannot adjudicate between the two explanations: cell of origin was assessable in only 50 patients, among whom non-GCB cases were numerically more frequent above every threshold but significant at none (at ≥50%, 56% versus 38%; *p* = 0.25).

In the prespecified model, IPI ≥ 3 was the only covariate independently associated with both endpoints, and it retained independence in all ten threshold models (adjusted HR, 2.05–2.46), mirroring real-world Turkish data [16]. This is more than a consistency check: the same cohort, events and specifications that detected no effect of PD-L1 recovered an effect of the IPI on every occasion. One qualification applies—when Ann Arbor stage was forced into the model the IPI was no longer independently associated with either endpoint, whereas the PD-L1 estimate was unchanged. The independence of the IPI is thus conditional on the prespecified specification; the PD-L1 null is not.

The distinction between prognostic and predictive value is relevant here. PD-L1 expression, particularly when driven by 9p24.1 alterations, predicts response to PD-1 blockade in PMBCL [11], and the same gene alterations that may worsen frontline outcomes can mark responsiveness at relapse [9]; PD-L1-directed strategies continue to be explored across large B-cell lymphomas [12]. Our findings indicate that in unselected DLBCL treated with chemoimmunotherapy, tumor-cell PD-L1 by TPS is unlikely to serve as a stand-alone prognostic marker. No patient in this cohort received pembrolizumab or any other checkpoint inhibitor at any point, reflecting the accrual period and the absence of an approved indication in DLBCL, so our data describe prognostic behavior under chemoimmunotherapy alone. Whether patients with a TPS ≥ 1%, or those above the higher thresholds examined, derive benefit from PD-1 blockade cannot be addressed by this design and remains open. The threshold behavior documented above may nonetheless transfer: any use of a TPS cut-off to select patients depends on the assay to a degree that the nominal value does not convey [22].

The specific contribution of this study is a direct, independent test of a previously published threshold rather than another survey of an unresolved marker. Follow-up was mature (median 89.8 months), the prespecified model satisfied the proportional-hazards assumption with an adequate events-per-variable ratio, and the contribution of PD-L1 beyond the IPI was tested directly rather than inferred. Every cut-off leaving at least eight patients per group is reported, so no threshold could have been chosen for its result.

Several limitations should be considered. First, the study is retrospective, single-center and small. With 95 patients, 38 deaths and a PD-L1-negative group of 22, it is underpowered to exclude a modest prognostic effect (OS adjusted HR, 0.73; 95% CI, 0.35–1.53), and this applies with particular force at the ≥70% threshold, where 25 patients and 13 deaths inform both the unadjusted association and our attribution of it to the IPI. A cohort of this size can address the question actually posed—whether a threshold specified in advance from an external report survives adjustment for the IPI—but not the discovery of a new one, and it cannot establish the absence of a small effect. Second, the cohort was large B-cell lymphoma rather than DLBCL-NOS alone: seven cases were provisionally classified as HGBL-NOS without fluorescence in situ hybridization, and therefore without genetic exclusion of *MYC* and *BCL2* rearrangements, and one was T-cell/histiocyte-rich. Both analyses were reproduced in the 87 DLBCL-NOS patients, but the caveats above about the heterogeneity of this family still apply. Third, the 22C3 assay was performed outside its validated platform, without independent external review. We regard this as a material weakness rather than a formality: our scores are not interchangeable with those of the companion-diagnostic assay, our positivity rates should not be compared directly with cohorts stained on the validated platform, and the thresholds examined would require re-testing on the validated platform before clinical use. Both pathologists scored each case jointly, so interobserver agreement could not be quantified; residual measurement error would bias hazard ratios toward the null by an unknown amount. Fourth, only tumor-cell TPS was evaluated, so microenvironmental, macrophage-associated and soluble PD-L1 were not assessed [10,14,21,25], and no patient received checkpoint blockade, so no predictive inference is possible. Fifth, cell-of-origin and double-expressor status were unavailable in about half the cases. Finally, all analyses beyond the prespecified ≥1% threshold were post hoc and no validation cohort was available; the formal test of incremental value was applied to OS only.

## 5. Conclusions

In this retrospective cohort of 95 patients with large B-cell lymphoma treated with frontline chemoimmunotherapy, tumor-cell PD-L1 expression assessed by the 22C3 TPS was not independently associated with OS (adjusted HR, 0.73; 95% CI, 0.35–1.53; *p* = 0.40) or PFS (adjusted HR, 0.91; 95% CI, 0.44–1.86; *p* = 0.79), and this held across five thresholds, as a continuous variable, and under a flexible spline model. An unadjusted association with OS emerged at the externally specified TPS ≥ 70% threshold (HR, 2.02; 95% CI, 1.02–4.02), but patients above this threshold more often carried an IPI ≥ 3, and the association did not survive adjustment for it. In the prespecified model, a high IPI (≥3) was the only covariate independently associated with both endpoints. These findings indicate that tumor-cell PD-L1 assessed by TPS did not add prognostic information beyond the IPI for overall survival, and that patients above the ≥70% threshold were enriched for higher IPI scores, with independent prognostic value not demonstrated after adjustment. Its role should be re-examined in larger, prospectively validated cohorts incorporating microenvironmental PD-L1 assessment and IPI-adjusted analysis.

## Figures and Tables

**Table 1:** Baseline characteristics of the cohort according to PD-L1 status.

Variable	PD-L1-Negative (*n* = 22)	PD-L1-Positive (*n* = 73)	Total (*n* = 95)	*p*
Age, years, median (IQR)	54.5 (46.5–62.0)	58.0 (49.0–67.0)	58.0 (48.0–65.5)	0.340
Sex, *n* (%)				0.519
Female	7 (32)	31 (42)	38 (40)	
Male	15 (68)	42 (58)	57 (60)	
Ann Arbor stage, *n* (%)				0.282
I–II	9 (41)	19 (26)	28 (29)	
III–IV	13 (59)	54 (74)	67 (71)	
B symptoms, present, *n* (%)	4 (18)	19 (26)	23 (24)	0.639
Extranodal involvement, yes, *n* (%)	21 (95)	60 (82)	81 (85)	0.177
Bone-marrow involvement, yes, *n* (%)	3 (14)	9 (12)	12 (13)	1.000
CNS involvement, yes, *n* (%)	2 (9)	3 (4)	5 (5)	0.327
Bulky disease, yes, *n* (%)	5 (23)	29 (40)	34 (36)	0.228
IPI ≥ 3, *n* (%)	8 (36)	35 (48)	43 (45)	0.476
LDH, U/L, median (IQR)	329.0 (245.0–382.0)	307.0 (245.0–382.0)	307.0 (245.0–382.0)	0.678
Cell of origin, *n* (%) †				1.000
GCB	7 (58)	21 (55)	28 (56)	
Non-GCB	5 (42)	17 (45)	22 (44)	
Double-expressor, yes, *n* (%) ‡	4 (36)	6 (13)	10 (18)	0.093
Ki-67 ≥ 80%, *n* (%)	13 (59)	36 (49)	49 (52)	0.575
R-CHOP alone, *n* (%)	17 (77)	54 (74)	71 (75)	0.974

IQR, interquartile range; CNS, central nervous system; IPI, International Prognostic Index; LDH, lactate dehydrogenase; GCB, germinal-center B-cell-like; R-CHOP, rituximab, cyclophosphamide, doxorubicin, vincristine, prednisone. Categorical variables were compared with the chi-square test with continuity correction, or with the Fisher exact test where any expected count was below five; continuous variables were compared with the Mann–Whitney U test. † Cell of origin assessable in 50 patients. ‡ Double-expressor status assessable in 56 patients.

**Table 2:** Univariable and multivariable Cox regression for overall and progression-free survival.

Variable	OS Univariable	OS Multivariable	PFS Univariable	PFS Multivariable
PD-L1-positive (≥1%)	0.86 (0.42–1.78), *p* = 0.693	0.73 (0.35–1.53), *p* = 0.404	1.05 (0.52–2.13), *p* = 0.890	0.91 (0.44–1.86), *p* = 0.792
Age (per year)	1.01 (0.99–1.03), *p* = 0.458	—	1.00 (0.98–1.02), *p* = 0.835	—
Male sex	0.98 (0.51–1.88), *p* = 0.955	—	0.87 (0.47–1.60), *p* = 0.655	—
Ann Arbor III–IV	5.24 (1.84–14.88), *p* = 0.002	—	13.07 (3.14–54.41), *p* < 0.001	—
B symptoms	1.10 (0.54–2.25), *p* = 0.792	—	1.41 (0.74–2.71), *p* = 0.297	—
Extranodal involvement	1.27 (0.49–3.25), *p* = 0.624	—	1.05 (0.44–2.49), *p* = 0.912	—
Bone-marrow involvement	2.97 (1.34–6.62), *p* = 0.008	—	2.26 (1.04–4.91), *p* = 0.040	—
Bulky disease	1.01 (0.52–1.98), *p* = 0.965	—	1.10 (0.59–2.05), *p* = 0.767	—
IPI ≥ 3	2.34 (1.22–4.51), *p* = 0.011	2.42 (1.24–4.72), *p* = 0.009	2.28 (1.22–4.23), *p* = 0.009	2.28 (1.22–4.29), *p* = 0.010
Ki-67 ≥ 80%	0.72 (0.38–1.37), *p* = 0.314	0.75 (0.39–1.43), *p* = 0.382	0.84 (0.46–1.53), *p* = 0.568	0.90 (0.49–1.64), *p* = 0.729
Non-GCB †	1.46 (0.66–3.21), *p* = 0.349	—	1.22 (0.59–2.50), *p* = 0.589	—
Double-expressor ‡	0.98 (0.36–2.69), *p* = 0.971	—	0.96 (0.36–2.55), *p* = 0.930	—

Values are hazard ratios (95% confidence intervals). The multivariable model included PD-L1 status, IPI, and Ki-67; dashes indicate variables not entered into the multivariable model. OS, overall survival; PFS, progression-free survival; IPI, International Prognostic Index; GCB, germinal-center B-cell-like. † Non-GCB assessable in 50 patients. ‡ Double-expressor status assessable in 56 patients.

**Table 3:** Post hoc analyses of PD-L1 across tumor proportion score definitions.

PD-L1 Definition	*n* Positive (%)	OS Log-Rank *p* (*q*)	OS Multivariable	PFS Log-Rank *p* (*q*)	PFS Multivariable
TPS ≥ 1% †	73 (77)	0.69 (0.87)	0.73 (0.35–1.53), *p* = 0.40 (*q* = 0.81)	0.89 (0.93)	0.91 (0.44–1.86), *p* = 0.79 (*q* = 0.99)
TPS ≥ 10%	68 (72)	0.93 (0.93)	0.87 (0.43–1.78), *p* = 0.70 (*q* = 0.99)	0.50 (0.87)	1.09 (0.54–2.19), *p* = 0.81 (*q* = 0.99)
TPS ≥ 25% ‡	59 (62)	0.53 (0.87)	0.70 (0.36–1.34), *p* = 0.28 (*q* = 0.81)	0.56 (0.87)	0.73 (0.40–1.36), *p* = 0.32 (*q* = 0.81)
TPS ≥ 50%	38 (40)	0.62 (0.87)	1.03 (0.53–1.98), *p* = 0.94 (*q* = 0.99)	0.59 (0.87)	1.00 (0.54–1.87), *p* = 0.99 (*q* = 0.99)
TPS ≥ 70% §	25 (26)	0.041 (0.30)	1.53 (0.74–3.18), *p* = 0.25 (*q* = 0.81)	0.061 (0.30)	1.42 (0.71–2.83), *p* = 0.32 (*q* = 0.81)
TPS, per 10-point increase ¶	—	NA	1.00 (0.90–1.11), *p* = 0.96	NA	1.00 (0.91–1.10), *p* = 0.97

Values in the multivariable columns are hazard ratios (95% confidence intervals). Each model included the PD-L1 term shown, IPI, and Ki-67 and was otherwise identical to the prespecified model in Table 2; diagnostics across the ten threshold models are given in Section 3.4. *p* values are nominal; *q* values are Benjamini–Hochberg-corrected within the family of ten threshold analyses (five thresholds × two endpoints), applied separately to the log-rank and multivariable *p* values. The full range of examined cut-offs is given in Supplementary Tables S1A and S1B. OS, overall survival; PFS, progression-free survival; TPS, tumor proportion score; IPI, International Prognostic Index; NA, not applicable. † Prespecified threshold; operationally equivalent to ≥5% (Section 3.2). ‡ Operationally equivalent to ≥30% (Section 3.2). § Specified from an external report [3] rather than from these data (Section 3.4). ¶ Modeled as a continuous variable; the hazard ratio per 10-point increment is 0.997 for OS and 1.002 for PFS before rounding, and this analysis is not included in the multiplicity correction (Section 2.8). NA, not applicable. The dash indicates that the number positive is not applicable, as TPS was modeled as a continuous variable.

**Table 4:** Incremental prognostic value of PD-L1 beyond the IPI for overall survival.

PD-L1 Term Added	LR χ^2^ (1 df)	*p*	ΔAIC	*C*-Index	Optimism-Corrected *C*
**Base model: IPI (ordinal score)**					
None (base model)	—	—	—	0.643	0.641
TPS ≥ 1%	0.77	0.38	+1.23	0.658	0.644
TPS ≥ 10%	0.21	0.65	+1.79	0.654	0.637
TPS ≥ 25%	1.17	0.28	+0.83	0.660	0.645
TPS ≥ 50%	0.00	0.99	+2.00	0.642	0.624
TPS ≥ 70%	1.03	0.31	+0.97	0.652	0.640
TPS, per 10-point increase	0.03	0.85	+1.97	0.649	0.629

The IPI was entered as its ordinal score (0–5) and each row adds the single PD-L1 term shown to the base model. LR χ^2^ is the likelihood ratio statistic against the base model on one degree of freedom; a positive ΔAIC indicates that the added term does not offset its cost in model complexity. *C*-indices were corrected for optimism as described in Section 2.8, and no PD-L1 model exceeded the base model by more than 0.004. The corresponding analysis with Ki-67 added to the base model, and with the IPI entered as the prespecified binary variable, is given in Supplementary Table S5; the conclusion was unchanged. LR, likelihood ratio; AIC, Akaike information criterion; TPS, tumor proportion score; IPI, International Prognostic Index. The shaded bold row is a heading, not a data row. Dashes indicate statistics that are not applicable to the base model, which is the comparator.

## Data Availability

The data presented in this study are available on request from the corresponding author due to institutional and ethical approval.

## Supplementary Materials

The following supporting information can be downloaded at https://www.mdpi.com/article/10.3390/diagnostics16183036/s1. Supplementary Methods: Full description of the post hoc, incremental-value and sensitivity analyses; Figure S1: Restricted cubic spline dose–response between PD-L1 tumor proportion score (TPS) and outcome; Figure S2: Unadjusted hazard ratios for PD-L1 across the full range of examined TPS cut-offs; Table S1A: Association between PD-L1 and overall survival across the full range of examined tumor proportion score cut-offs; Table S1B: Association between PD-L1 and progression-free survival across the full range of examined tumor proportion score cut-offs; Table S2: Baseline characteristics according to the PD-L1 tumor proportion score 70% cut-off; Table S3A: Sensitivity analyses for overall survival in the DLBCL-NOS and R-CHOP subgroups; Table S3B: Sensitivity analyses for progression-free survival in the DLBCL-NOS and R-CHOP subgroups; Table S4: Baseline laboratory parameters according to PD-L1 status; Table S5: Incremental prognostic value of PD-L1 beyond the IPI with Ki-67 in the base model, for overall survival.
