# Supplementary material for: Is There a Tumor Proportion Score Threshold at Which Tumor-Cell PD-L1 Adds Prognostic Information Beyond the International Prognostic Index in Large B-Cell Lymphoma?
Source: Diagnostics (Basel). 2026 Sep 19;16(18):3036. doi: 10.3390/diagnostics16183036 (PMC13605719; doi:10.3390/diagnostics16183036)
Supplement: Supplementary file 1 [file diagnostics-16-03036-s001.zip › diagnostics-4492613-supplementary.pdf]

Supplementary Materials

Is There a Tumor Proportion Score Threshold at Which Tumor-Cell PD-L1 Adds Prognostic Information Beyond the International Prognostic Index in Large B-Cell Lymphoma?

Mehmet Mutlu Kidi, Suheda Atas Ipek, Sendag Yaslikaya, Mahmut Buyuksimsek, Mehmet Turker, Yasemin Aydinalp Camadan, Sedat Biter, Tolga Koseci, Tugba Toyran, Melek Ergin, Berksoy Sahin, Ismail Oguz Kara, Ertugrul Bayram

Supplementary Methods

Because the prognostic value of PD-L1 has been reported to vary with the threshold applied [3,15], the following post hoc analyses were performed. First, the Kaplan–Meier, log-rank, and multivariable Cox analyses described above were repeated in full at TPS thresholds of ≥10%, ≥25%, ≥50%, and ≥70%, using the same covariates as the prespecified model. This yielded a family of ten threshold analyses (five thresholds × two endpoints), within which the log-rank and the multivariable PD-L1 P values were separately corrected for multiplicity by the Benjamini–Hochberg procedure; corrected values are reported as q. Because the ≥70% threshold was specified from an external report rather than from these data [3], its uncorrected P value is also reported. Where an unadjusted and an adjusted estimate diverged, covariates were added to the model one at a time to identify the source of the attenuation. To document that no threshold was selected on the basis of its result, every cut-off leaving at least eight patients in each group was additionally examined; these results are presented in Supplementary Tables S1A and S1B and Supplementary Figure S2 and were not used for inference. Second, TPS was modeled as a continuous variable, with HRs expressed per 10-point increment. Third, to allow for a non-linear dose-response, TPS was modeled with a restricted cubic spline using three knots placed at the 10th, 50th, and 90th percentiles of the observed distribution (0%, 40%, and 90%); the overall association and departure from linearity were assessed by likelihood ratio tests against models without TPS and with linear TPS, respectively. Models specified with four or five knots did not converge and were not pursued. The continuous and spline analyses address dose-response rather than threshold selection and were therefore reported separately from, and not corrected within, the threshold family.

The prognostic contribution of PD-L1 beyond established clinical risk was assessed directly. The IPI was modeled both as the prespecified binary variable (≥3) and as its ordinal score, since dichotomizing an ordinal index can leave residual confounding. PD-L1 terms were then added to a base model containing the IPI alone and, as a sensitivity analysis, to a base model containing the IPI together with Ki-67, and the incremental contribution was quantified by the likelihood ratio test, the change in the Akaike information criterion (AIC), and the change in Harrell's C-index. This analysis was restricted to OS, the primary endpoint. Because apparent discrimination is optimistically biased in the sample used to fit the model, C-indices were also corrected for optimism by bootstrapping with 500 resamples under a fixed random seed; the corrected values are interpreted only as evidence for or against a material gain in discrimination, not as a ranking of models.

Further sensitivity analyses were performed. The prespecified TPS ≥1% analysis and the externally specified TPS ≥70% analysis were repeated in the 87 patients with DLBCL-NOS and, separately, in the 71 patients treated with R-CHOP alone, to test whether histological or treatment heterogeneity influenced the PD-L1 estimates. Ann Arbor stage was forced into the multivariable model to test whether its prespecified exclusion influenced the PD-L1 estimate. No data-driven search for an optimal cut-off was undertaken: in the absence of an independent validation cohort, a cut-off selected to maximize a test statistic in the same cohort would not be interpretable, and any resulting threshold would require external validation before use. The prespecified analyses were performed in IBM SPSS Statistics v25 (IBM Corp., Armonk, NY, USA). The post hoc threshold, continuous, spline, and multiplicity analyses were performed in Python 3.12 using the lifelines (v0.30.3), statsmodels, and SciPy packages.

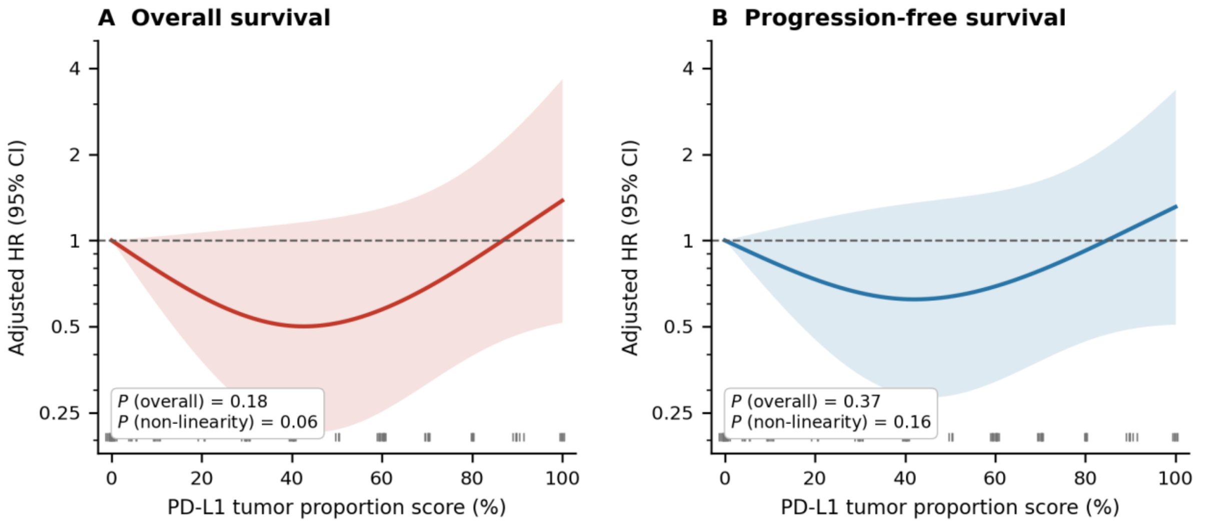

Figure S1. Restricted cubic spline dose-response between PD-L1 tumor proportion score (TPS) and outcome, for (A) overall survival and (B) progression-free survival. Models were adjusted for the International Prognostic Index (IPI) and Ki-67, with three knots placed at the 10th, 50th, and 90th percentiles of the observed TPS distribution (0%, 40%, and 90%). Hazard ratios are plotted relative to a TPS of 0%; shaded bands are 95% CIs and the dashed line marks HR = 1. Tick marks along the lower margin show the observed TPS values, horizontally jittered. *P* (overall) tests the association between TPS and outcome; *P* (non-linearity) tests departure from a linear dose-response. Models specified with four or five knots did not converge and are not shown.

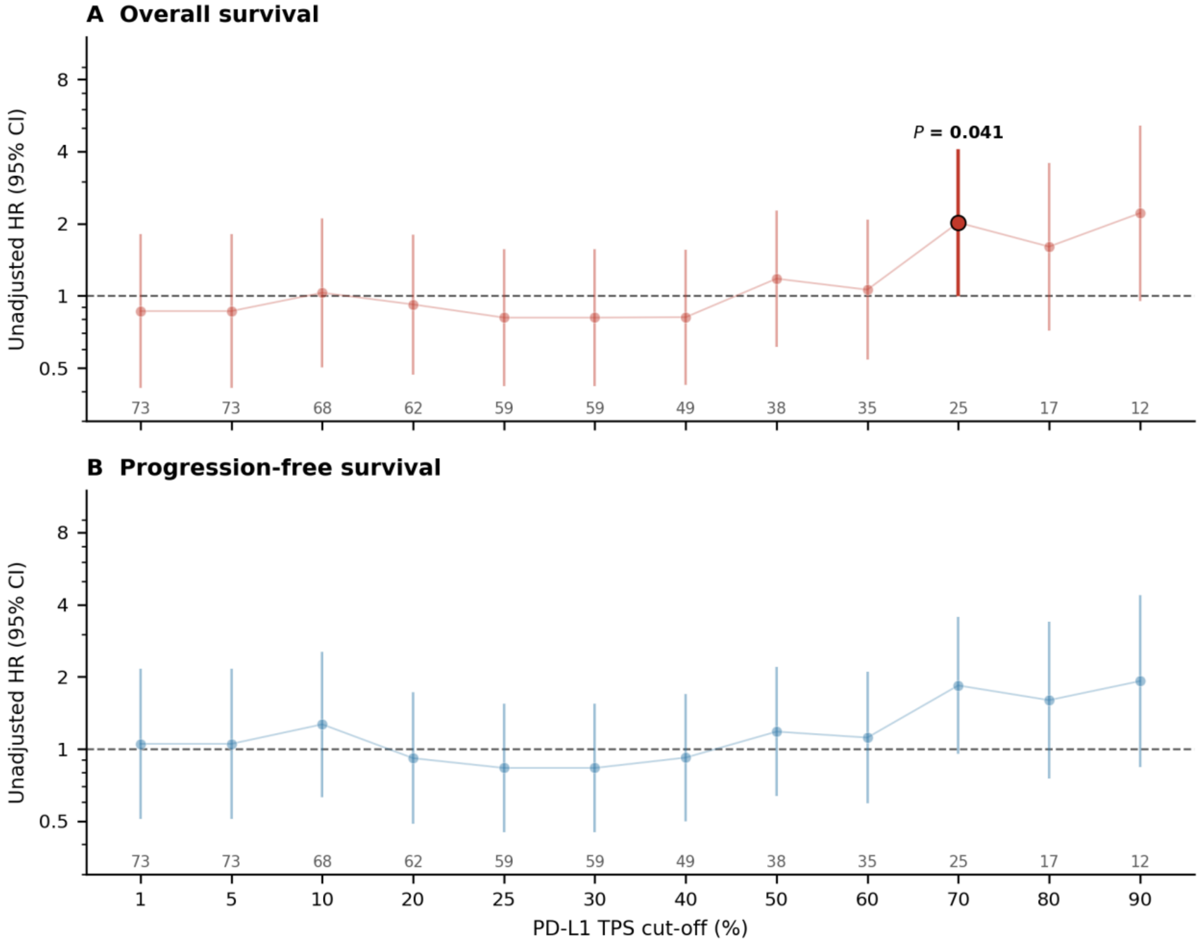

Figure S2. Unadjusted hazard ratios for PD-L1 across the full range of examined TPS cut-offs, for (A) overall survival and (B) progression-free survival. Every cut-off leaving at least eight patients in each group is shown; ≥100% (n = 5 positive) was therefore not examined. Points are unadjusted hazard ratios with 95% CIs; the dashed line marks HR = 1. Solid, black-outlined points denote a nominal log-rank $P < 0.05$; faded points denote $P \geq 0.05$. The numbers along the bottom of each panel are the

counts of PD-L1-positive patients at that cut-off. Cut-offs are placed at equally spaced positions; the horizontal axis is an ordered list of the cut-offs examined, not a numerical scale. These analyses are presented for transparency, to document that no threshold was selected on the basis of its result; they were not used for inference and were not corrected for multiplicity.

Table S1A. Association between PD-L1 and overall survival across the full range of examined tumor proportion score cut-offs.

TPS cut-off	n positive (%)	OS events in the TPS-positive group, n	log-rank *P*	Unadjusted HR (95% CI)	Adjusted HR (95% CI)	Adjusted *P*
≥1%	73 (77)	28	0.694	0.86 (0.42–1.78)	0.73 (0.35–1.53)	0.404
≥5% †	73 (77)	28	0.694	0.86 (0.42–1.78)	0.73 (0.35–1.53)	0.404
≥10%	68 (72)	27	0.932	1.03 (0.51–2.08)	0.87 (0.43–1.78)	0.703
≥20%	62 (65)	24	0.807	0.92 (0.48–1.78)	0.81 (0.42–1.59)	0.545
≥25%	59 (62)	22	0.526	0.81 (0.43–1.55)	0.70 (0.36–1.34)	0.279
≥30% ‡	59 (62)	22	0.526	0.81 (0.43–1.55)	0.70 (0.36–1.34)	0.279
≥40%	49 (52)	18	0.528	0.81 (0.43–1.54)	0.67 (0.35–1.28)	0.225
≥50%	38 (40)	16	0.615	1.18 (0.62–2.25)	1.03 (0.53–1.98)	0.936
≥60%	35 (37)	14	0.860	1.06 (0.55–2.05)	0.99 (0.51–1.92)	0.968
**≥70%**	**25 (26)**	**13**	**0.041**	**2.02 (1.02–4.02)**	**1.53 (0.74–3.18)**	**0.252**
≥80%	17 (18)	8	0.237	1.60 (0.73–3.54)	1.33 (0.60–2.97)	0.487
≥90%	12 (13)	7	0.054	2.21 (0.96–5.07)	1.83 (0.79–4.26)	0.158

All cut-offs leaving at least eight patients in each group are shown; ≥100% (n = 5 positive) was therefore not examined. “Events” is the number of events occurring among PD-L1-positive patients at that cut-off. Adjusted models included PD-L1, the IPI, and Ki-67, and were otherwise identical to the prespecified model in Table 2 of the main text. The ≥70% row is shaded because that threshold was specified from an external report and is reported in the main text; across both endpoints it was the only cut-off at which the log-rank *P* value fell below 0.05. These analyses are presented for transparency, to document that no threshold was selected on the basis of its result; they were not used for inference and were not corrected for multiplicity. † No case was scored between 1% and 4%, so ≥5% is identical to ≥1%. ‡ No case was scored between 21% and 29%, so ≥30% is identical to ≥25%. HR, hazard ratio; CI, confidence interval; TPS, tumor proportion score; IPI, International Prognostic Index. Thirty-eight deaths occurred in the full cohort.

Table S1B. Association between PD-L1 and progression-free survival across the full range of examined tumor proportion score cut-offs.

TPS cut-off	n positive (%)	PFS events in the TPS-positive group, n	log-rank *P*	Unadjusted HR (95% CI)	Adjusted HR (95% CI)	Adjusted *P*
≥1%	73 (77)	33	0.890	1.05 (0.52–2.13)	0.91 (0.44–1.86)	0.792
≥5% †	73 (77)	33	0.890	1.05 (0.52–2.13)	0.91 (0.44–1.86)	0.792
≥10%	68 (72)	32	0.497	1.27 (0.64–2.52)	1.09 (0.54–2.19)	0.805
≥20%	62 (65)	27	0.780	0.92 (0.49–1.70)	0.83 (0.45–1.56)	0.567
≥25%	59 (62)	25	0.557	0.83 (0.45–1.53)	0.73 (0.40–1.36)	0.325
≥30% ‡	59 (62)	25	0.557	0.83 (0.45–1.53)	0.73 (0.40–1.36)	0.325
≥40%	49 (52)	21	0.788	0.92 (0.51–1.68)	0.78 (0.42–1.43)	0.418
≥50%	38 (40)	18	0.589	1.18 (0.64–2.17)	1.00 (0.54–1.87)	0.988
≥60%	35 (37)	16	0.728	1.12 (0.60–2.07)	1.00 (0.54–1.87)	0.994
**≥70%**	**25 (26)**	**14**	**0.061**	**1.84 (0.96–3.52)**	**1.42 (0.71–2.83)**	**0.323**
≥80%	17 (18)	9	0.211	1.60 (0.76–3.35)	1.30 (0.61–2.78)	0.492

TPS cut-off	n positive (%)	PFS events in the TPS-positive group, n	log-rank *P*	Unadjusted HR (95% CI)	Adjusted HR (95% CI)	Adjusted *P*
≥90%	12 (13)	7	0.110	1.92 (0.85–4.33)	1.56 (0.68–3.57)	0.292

All cut-offs leaving at least eight patients in each group are shown; ≥100% (n = 5 positive) was therefore not examined. “Events” is the number of events occurring among PD-L1-positive patients at that cut-off. Adjusted models included PD-L1, the IPI, and Ki-67, and were otherwise identical to the prespecified model in Table 2 of the main text. The ≥70% row is shaded because that threshold was specified from an external report and is reported in the main text; across both endpoints it was the only cut-off at which the log-rank *P* value fell below 0.05. These analyses are presented for transparency, to document that no threshold was selected on the basis of its result; they were not used for inference and were not corrected for multiplicity. † No case was scored between 1% and 4%, so ≥5% is identical to ≥1%. ‡ No case was scored between 21% and 29%, so ≥30% is identical to ≥25%. HR, hazard ratio; CI, confidence interval; TPS, tumor proportion score; IPI, International Prognostic Index. Forty-four progression-free survival events occurred in the full cohort.

Table S2. Baseline characteristics according to the PD-L1 tumor proportion score 70% cut-off.

Characteristic	TPS <70% (n = 70)	TPS ≥70% (n = 25)	*P*	Assessable, n
Age, years, median (IQR)	57 (43.8–65)	60 (53–70)	0.067	
LDH, U/L, median (IQR)	307.5 (245–382)	307 (258–382)	0.495	
IPI score, median (IQR)	2 (1–3)	3 (2–3)	<0.001	
Male sex, n (%)	44 (63)	13 (52)	0.353	
IPI ≥3, n (%)	25 (36)	18 (72)	0.002	
Ann Arbor stage III–IV, n (%)	45 (64)	22 (88)	0.039	
B symptoms, n (%)	16 (23)	7 (28)	0.597	
Bulky disease ≥7.5 cm, n (%)	24 (34)	10 (40)	0.633	
Bone-marrow involvement, n (%)	9 (13)	3 (12)	1.000	
CNS involvement, n (%)	5 (7)	0 (0)	0.321	
Ki-67 ≥80%, n (%)	39 (56)	10 (40)	0.244	
Non-GCB by Hans algorithm, n (%)	15 (41)	7 (54)	0.520	50
Double-expressor, n (%)	8 (21)	2 (12)	0.706	56
DLBCL-NOS, n (%)	63 (90)	24 (96)	0.676	
R-CHOP alone, n (%)	54 (77)	17 (68)	0.424	

Categorical variables were compared with the Fisher exact test and continuous variables with the Mann–Whitney U test. Where a variable was not assessable in all 95 patients, the number assessable is given in the last column. This table supports the analysis in Section 3.4 of the main text: patients above the 70% threshold more often had a high IPI and advanced stage, whereas the remaining characteristics did not differ. Among the 50 patients with assessable cell of origin, the proportion of non-GCB cases was also numerically higher above a tumor proportion score of 50% (56% versus 38%; odds ratio, 2.08; *P* = 0.25, Fisher exact), the comparison cited in the Discussion. IQR, interquartile range; LDH, lactate dehydrogenase; IPI, International Prognostic Index; CNS, central nervous system; GCB, germinal-center B-cell-like; DLBCL-NOS, diffuse large B-cell lymphoma, not otherwise specified; R-CHOP, rituximab, cyclophosphamide, doxorubicin, vincristine, prednisone; TPS, tumor proportion score.

Table S3A. Sensitivity analyses for overall survival in the DLBCL-NOS and R-CHOP subgroups.

PD-L1	Subgroup	n	n positive (%)	Total events, n	log-rank *P*	Unadjusted HR (95% CI)	Adjusted HR (95% CI)	Adjusted *P*
TPS ≥1%	Full cohort	95	73 (77)	38	0.694	0.86 (0.42–1.78)	0.73 (0.35–1.53)	0.404

PD-L1	Subgroup	n	n positive (%)	Total events, n	log-rank *P*	Unadjusted HR (95% CI)	Adjusted HR (95% CI)	Adjusted *P*
**TPS ≥1%**	**DLBCL-NOS only**	**87**	**66 (76)**	**34**	**0.750**	**0.88 (0.41–1.90)**	**0.74 (0.34–1.60)**	**0.444**
TPS ≥1%	R-CHOP alone	71	54 (76)	29	0.968	1.02 (0.43–2.39)	0.88 (0.37–2.09)	0.770
TPS ≥1%	DLBCL-NOS + R-CHOP	65	49 (75)	25	0.959	1.02 (0.41–2.57)	0.89 (0.35–2.28)	0.816
TPS ≥70%	Full cohort	95	25 (26)	38	0.041	2.02 (1.02–4.02)	1.53 (0.74–3.18)	0.252
**TPS ≥70%**	**DLBCL-NOS only**	**87**	**24 (28)**	**34**	**0.012**	**2.43 (1.18–4.97)**	**1.67 (0.78–3.59)**	**0.189**
TPS ≥70%	R-CHOP alone	71	17 (24)	29	0.086	2.00 (0.89–4.48)	1.33 (0.54–3.26)	0.534
TPS ≥70%	DLBCL-NOS + R-CHOP	65	17 (26)	25	0.040	2.36 (1.01–5.50)	1.35 (0.53–3.41)	0.525

The cohort comprised 87 patients with DLBCL-NOS, one with T-cell/histiocyte-rich large B-cell lymphoma, and seven with high-grade B-cell lymphoma, not otherwise specified; 71 patients received R-CHOP. Adjusted models included PD-L1, the IPI, and Ki-67, and were otherwise identical to the prespecified model in Table 2 of the main text. Only the prespecified TPS ≥1% threshold and the externally specified TPS ≥70% threshold were repeated in these subgroups (Section 2.8). The DLBCL-NOS rows are shaded. *P* values are nominal and were not corrected for multiplicity. HR, hazard ratio; CI, confidence interval; TPS, tumor proportion score; IPI, International Prognostic Index; DLBCL-NOS, diffuse large B-cell lymphoma, not otherwise specified. At the prespecified ≥1% threshold the estimate was unchanged in every subgroup. At ≥70% the unadjusted association was not diluted by restricting the cohort, but the adjusted estimate remained non-significant throughout.

Table S3B. Sensitivity analyses for progression-free survival in the DLBCL-NOS and R-CHOP subgroups.

PD-L1	Subgroup	n	n positive (%)	Total events, n	log-rank *P*	Unadjusted HR (95% CI)	Adjusted HR (95% CI)	Adjusted *P*
TPS ≥1%	Full cohort	95	73 (77)	44	0.890	1.05 (0.52–2.13)	0.91 (0.44–1.86)	0.792
**TPS ≥1%**	**DLBCL-NOS only**	**87**	**66 (76)**	**40**	**0.800**	**1.10 (0.52–2.32)**	**0.94 (0.44–1.99)**	**0.867**
TPS ≥1%	R-CHOP alone	71	54 (76)	33	0.642	1.22 (0.53–2.82)	1.06 (0.46–2.49)	0.885
TPS ≥1%	DLBCL-NOS + R-CHOP	65	49 (75)	29	0.608	1.27 (0.51–3.12)	1.12 (0.45–2.77)	0.815
TPS ≥70%	Full cohort	95	25 (26)	44	0.061	1.84 (0.96–3.52)	1.42 (0.71–2.83)	0.323
**TPS ≥70%**	**DLBCL-NOS only**	**87**	**24 (28)**	**40**	**0.022**	**2.14 (1.10–4.17)**	**1.51 (0.73–3.09)**	**0.266**
TPS ≥70%	R-CHOP alone	71	17 (24)	33	0.171	1.71 (0.79–3.73)	1.08 (0.46–2.54)	0.861
TPS ≥70%	DLBCL-NOS + R-CHOP	65	17 (26)	29	0.100	1.94 (0.87–4.35)	1.04 (0.43–2.49)	0.929

The cohort comprised 87 patients with DLBCL-NOS, one with T-cell/histiocyte-rich large B-cell lymphoma, and seven with high-grade B-cell lymphoma, not otherwise specified; 71 patients received R-CHOP. Adjusted models included PD-L1, the IPI, and Ki-67, and were otherwise identical to the prespecified model in Table 2 of the main text. Only the prespecified TPS ≥1% threshold and the externally specified TPS ≥70% threshold were repeated in these subgroups (Section 2.8). The DLBCL-NOS rows are shaded. *P* values are nominal and were not corrected for multiplicity. HR, hazard ratio; CI, confidence interval; TPS, tumor proportion score; IPI, International Prognostic Index; DLBCL-NOS, diffuse large B-cell lymphoma, not otherwise specified. The pattern matched that for overall survival: the adjusted estimate was non-significant in every subgroup at both thresholds.

Table S4. Baseline laboratory parameters according to PD-L1 status.

Variable	PD-L1 negative (n = 22)	PD-L1 positive (n = 73)	Total (n = 95)	P
Hemoglobin, g/dL	12.0 (10.4–13.2)	12.0 (9.8–13.5)	12.0 (9.9–13.5)	0.961
Albumin, g/dL	3.5 (2.9–3.9)	3.5 (3.1–3.9)	3.5 (3.0–3.9)	0.311

Leukocytes, $\times 10^3$/µL	8.8 (6.2–9.4)	7.8 (6.3–9.3)	8.1 (6.2–9.3)	0.466
Absolute lymphocyte count, /µL	1791 (1000–2267.5)	1800 (1000–2120)	1800 (1000–2160)	0.710
Absolute neutrophil count, /µL	4150 (2800–5840)	4650 (2800–6000)	4500 (2800–5840)	0.545
Platelets, $\times 10^3$/µL	334 (257–394.2)	311 (264–362)	311 (259–362)	0.996
β2-microglobulin, mg/L	3.4 (2.0–4.8)	2.8 (2.3–4.6)	2.8 (2.2–4.6)	0.808
CRP, mg/L	6.0 (3.0–18.9)	6.0 (2.4–22.0)	6.0 (3.0–22.0)	0.828

All values are median (interquartile range), compared with the Mann–Whitney U test. None of these parameters entered the multivariable models. CRP, C-reactive protein.

Table S5. Incremental prognostic value of PD-L1 beyond the IPI with Ki-67 in the base model, for overall survival.

**PD-L1 term added**	**LR χ^2 (1 df)**	**P**	**ΔAIC**	**C-index**	**Optimism-corrected C**
None (base model)	—	—	—	0.653	0.637
TPS ≥1%	0.73	0.39	+1.27	0.663	0.636
TPS ≥10%	0.21	0.65	+1.79	0.657	0.627
TPS ≥25%	1.19	0.27	+0.81	0.662	0.634
TPS ≥50%	0.00	0.95	+2.00	0.653	0.624
TPS ≥70%	0.88	0.35	+1.12	0.657	0.631
TPS, per 10-point increase	0.04	0.83	+1.96	0.654	0.622

Base model: IPI (ordinal score) + Ki-67. Sensitivity analysis corresponding to Table 4 of the main text; the conclusion was unchanged. With the IPI entered as the prespecified binary variable (≥3) alongside Ki-67, likelihood ratio P = 0.26–0.96, ΔAIC +0.73 to +2.00, and corrected C-index 0.608–0.624 versus 0.628 for the base model. LR, likelihood ratio; AIC, Akaike information criterion; TPS, tumor proportion score; IPI, International Prognostic Index.
